# Evolutionary expansion and functional diversification of oligopeptide transporter gene family in rice

**DOI:** 10.1186/1939-8433-5-12

**Published:** 2012-06-22

**Authors:** Tao Liu, Jiqing Zeng, Kuaifei Xia, Tian Fan, Yuge Li, Yaqin Wang, Xinlan Xu, Mingyong Zhang

**Affiliations:** 1 Key Laboratory of South China Agricultural Plant Genetics and Breeding, South China Botanical Garden, Chinese Academy of Sciences, Guangzhou, 510650 People's Republic of China; 2 Graduate University of Chinese Academy of Sciences, Beijing, 100049 People's Republic of China; 3 Key Laboratory of Plant Resources Conservation and Sustainable Utilization, South China Botanical Garden, Chinese Academy of Sciences, Guangzhou, 510650 People's Republic of China; 4 Guangdong Key Lab of Biotechnology for Plant Development, College of Life Science, South China Normal University, Guangzhou, 510631 People's Republic of China

**Keywords:** Oligopeptide transporters, Rice, Evolutionary expansion, Expression profile

## Abstract

**Background:**

Oligopeptide transporters (*OPTs*) play important roles in the mobilization of organic nitrogenous compounds and usually associate with tissues that show signs of rapid protein hydrolysis, such as germinating seeds and senescing leaves. This study is to investigate rice *OPT* genes.

**Results:**

A total of sixteen *OsOPT* genes (*Os* for *Oryza sative* L.) were identified in the rice genome, which were then classified into six sections that belong to two subfamilies (the PT and YSL subfamily). The major mechanisms for evolutionary expansion of the sixteen genes during the rice genome evolution include segmental and tandem duplication. Calculation of the duplication event dates indicated that the sixteen genes originated from nine original *OsOPT* genes, and the duplication events could be classified into three evolutionary stages. The first evolutionary stage occurred approximately 50 million years ago (Mya) and involved the evolution of four new genes. The second evolutionary stage was approximately 20 Mya and was marked by the appearance of two new genes, and the third evolutionary stage was approximately 9 Mya when two new genes evolved. Mining of the expression database and RT-PCR analysis indicated that the expression of most duplicated *OsOPT* genes showed high tissue specificities. Diverse expression patterns for the sixteen genes were evaluated using both semi-quantitative RT-PCR and the MPSS data. Expression levels of some *OsOPT* genes were regulated by abiotic and biotic stresses suggesting the potential involvement of these gene products in rice stress adaptation. Five *OsOPT* gene mutants showed abnormal development and growth, the primary analysis of five *OsOPT* gene mutants suggested that they may be necessary for rice development.

**Conclusions:**

These results suggested that rice-specific *OsOPT* genes might be potentially useful in improving rice.

**Electronic supplementary material:**

The online version of this article (doi:10.1186/1939-8433-5-12) contains supplementary material, which is available to authorized users.

## Background

The acquisition and allocation of nitrogenous compounds are essential processes in plant growth and development (Williams and Miller [2001]). Peptide transport has been demonstrated to play important roles in these processes and is a commonly observed physiological phenomenon in both prokaryotes and eukaryotes (Higgins and Payne [1981]). Peptide transporters are characterized by the ability to transport a range in different types of peptides and their derivatives across membranes in an energy-dependent manner (Lubkowitz et al. [1997]; Lubkowitz et al. [1998]). The plant peptide transporters generally belong to three different gene families: the ABC-type transporter family, the peptide transporter family (PTR) and the oligopeptide transporter family (OPT) (Ouyang et al. [2010]; Rentsch et al. [2007]; Zhao et al. [2010]), each of which recognizes peptides of a specific length. In this study, we focused on the rice OPT family. The OPT family was first characterized in yeast (Lubkowitz et al. [1997]). Several other *OPT* genes have also been identified from other species, such as *CaOPT1* from *Candida albicans* *SpIsp4* from *Schizosaccharomyces pombe,* and *ScOPT2* from *Schizosaccharomyces cerevisiae* (Lubkowitz et al. [1998]). However, to date the OPT family has only been found in fungi, bacteria, plants and archaea, but not in animals (Lubkowitz [2006]).

The *OPT* genes have been classified into two subfamilies by phylogenetic analysis: the PT and the YSL subfamily (Curie et al. [2001]). Most of the information regarding the PT subfamily in plants comes from the study of *Arabidopsis*. Nine orthologs of the PT subfamily were identified in *Arabidopsis* and were named sequentially from *AtOPT1* to *AtOPT9*. Five (*AtOPT1, 4, 5, 6,* and *7*) of the orthologs were found to be functional tetra- and penta-peptide transporters by yeast complementation assay (Koh et al. [2002]; Osawa et al. [2006]). However, reduced *AtOPT3* expression resulted in decreased accumulation of iron in seeds while high levels resulted in the accumulation of iron in other tissues, suggesting that *AtOPT3* functions to maintain whole-plant iron homeostasis and iron nutrition of developing seeds (Stacey et al. [2007]). AtOPT6 proteins have the ability to transport glutathione derivatives (Cagnac et al. [2004]) and mammalian signaling peptides up to 10 amino acids in length (Pike et al. [2009]). *AtOPT* promoter-GUS fusion analysis revealed differential expression of 8 *AtOPT* genes: 6 *AtOPT* genes (*AtOPT1* *AtOPT3* *AtOPT4* *AtOPT6* *AtOPT7* and *AtOPT8*) were strongly expressed in vascular tissues, 3 (*AtOPT1* *AtOPT3* and *AtOPT8*) were uniquely expressed in pollens and one (*AtOPT6*) was expressed in ovules (Stacey et al. [2005]). Several *OPT* genes were also identified from other plants. *BjGT1* from *B*. *juncea* was found to transport glutathione and has been implicated in mediating heavy-metal toxicity (Bogs et al. [2003]).

The YSL subfamily is usually considered to transport the metals (Inoue et al. [2008]; Lee et al. [2009]). Iron (Fe) is an important micronutrient for living organisms (Bashir et al. [2010]). Maize yellow *stripe1* (*ZmYS1*) from *Zea mays* was demonstrated as a phytosiderophore transporter (Curie et al. [2001]; Roberts et al. [2004]; Schaaf [2004]). In rice, *OsYSL2* (Koike et al. [2004]), *OsYSL15* (Inoue et al. [2008]; Lee et al. [2009]) and *OsYSL18* (Aoyama et al. [2009]) are similar to orthologs of *ZmYSL1* in that they all have the ability to transport metal-nicotianamine. However, some members (*OsOPT1* *OsOPT3* *OsOPT4* *OsOPT5* and *OsOPT7*) of the PT subfamily in rice have been demonstrated to transport nicotianamine-bound iron (Vasconcelos et al. [2008]).

The roles of the *OPT* genes in plants, especially in rice, are still under investigation. To the best of our knowledge, no biofunctional data regarding the Os*OPT* genes are currently available. In eukaryotes, functional diversification follows the evolutionary expansion of a gene family. The evolutionary expansion of a gene family follows duplication events of an individual gene, the chromosomal segment, or the whole genome (Lynch and Conery [2000]; Moore and Purugganan [2005]; Xue and Fu [2008]). In this study, we identified the evolutionary expansion of the rice OsOPT family and showed function diversification of the duplicated genes by expression analysis and primary analysis of the mutants.

## Results

### Identification of 16 *OsOPT* gene homologues in the rice genome

A total of 17 putative OsOPT proteins have been identified from the rice genome database by BLASTP searching. However, one locus (LOC_Os02g46860) encodes a short protein (245aa) that was not found in NCBI; therefore, we considered it to be a pseudogene and did not analyze it in this study. The predicated OsOPT proteins were analyzed for Pfam matches in the Pfam database to confirm the 16 putative *OsOPT* genes (Table 1) as oligopeptide transporter-like homologues and to identify their domains. The analysis showed that all 16 putative *OsOPT* genes were homologous to the OPT family and their predicated proteins contained the SPYxEVRxxVxxxDDP domain, which is also found in other OPT proteins (Lubkowitz et al. [1998]; Wiles et al. [2006]). Next, we analyzed the parameters and the number of transmembrane helices of OsOPT proteins and found that the OsOPT proteins usually contain 12-16 transmembrane helices with molecular weights that range from 50 to 90 KDa. These parameters were similar to the description of the OPT family in the Pfam database.

**Table 1:** **Basic information of sixteen rice** ***OsOPT*** **genes**

Locus ID^a^	Protein					FL-cDNA^c^	Identity/similarity (E-value) to SpISP4	Putative Substrate^d^	Phenotypes of the mutant^e^
	Name	Length (aa)	MW (KDa)	TM^b^	pI				
LOC_Os01g43940	OsOPT1	755	83.60	16	8.92	NA	37/57%(3.1e-140)	Iron-chelate	
LOC_Os02g43370	OsYSL2	674	73.34	15	9.03	AB126253	19/36%(0.00099)	Iron-chelate	Dwarf, palegreen leaf, low tillerring, long clum, late heading
LOC_Os02g43410	OsYSL15	672	73.15	14	9.20	AB190923	22/41%(0.00019)	Iron-chelate	
LOC_Os02g46850	OsOPT8	559	56.23	11	8.59	AK071310	39/58%(1.7e-107)		
LOC_Os03g54000	OsOPT7	757	83.45	16	9.18	AK102404	37/57%(7.3e-123)	Iron-chelate	
LOC_Os04g44300	OsYSL13	688	78.77	14	9.10	AK067235	21/37%(0.0083)		
LOC_Os04g44320	OsYSL12	717	77.69	12	8.96	AK069437	21/37%(0.019)		Dwarf, short panicle, lesion leaf, low tillering
LOC_Os04g45860	OsYSL9	657	74.26	14	8.50	AK120923	21/38%(0.0023)		
LOC_Os04g45900	OsYSL16	675	73.81	14	9.30	AK070304	21/39%(0.013)		
LOC_Os04g50820	OsOPT6	737	82.25	15	8.86	AK070801	35/57%(3.5e-139)		
LOC_Os04g57840	OsYSL10	686	74.65	14	9.06	AK069645	21/39%(0.011)		
LOC_Os06g03540	OsOPT2	761	85.82	14	5.95	NA	37/37%(2.0e-145)		
LOC_Os06g03560	OsOPT3	766	86.14	12	6.53	AF393848	36/55%(1.7e-139)	Iron-chelate/GSH	Dwarf, glabrous leaf, semi-short clum, white belly rice kernel
LOC_Os06g03700	OsOPT4	760	85.10	12	7.87	AK072617	38/58%(2.0e-145)	Iron-chelate	Yellow-green plant, late heading, semi-dwarf, low tillerring
LOC_Os08g23130	OsOPT5	733	81.63	12	9.15	AK121257	35/55%(5.4e-132)	Iron-chelate	
LOC_Os08g38400	OsOPT9	752	82.82	14	9.17	AK100814	37/55%(5.1e-131)		Semi-dwarf, low tillering, semi-sterile pollen

^a^Locus ID was adopted from the MSU Rice Genome Annotation Project Database; ^b^Number of transmembrane domains; ^c^Corresponding full-length cDNA in GenBank; ^d^Putative substrate come from former publications. The NA indicates that this gene has no mRNA record in GenBank.^e^The phenotypes of the inserted mutants of *OsOPTs* except of *OsOPT9* were from different mutant database (*OsYSL2*, *OsYSL12*, and *OsOPT3* from Rice *Tos17* Insertion Mutant Database: http://tos.nias.affrc.go.jp/index.html.en, *OsOPT4* from Oryza Tag Line: http://urgi.versailles.inra.fr/OryzaTagLine/cgi-bin/focus_mutant.pl).

### Phylogenetic relationship, chromosomal location and gene structures of the *OsOPT* genes

**Figure 1:**
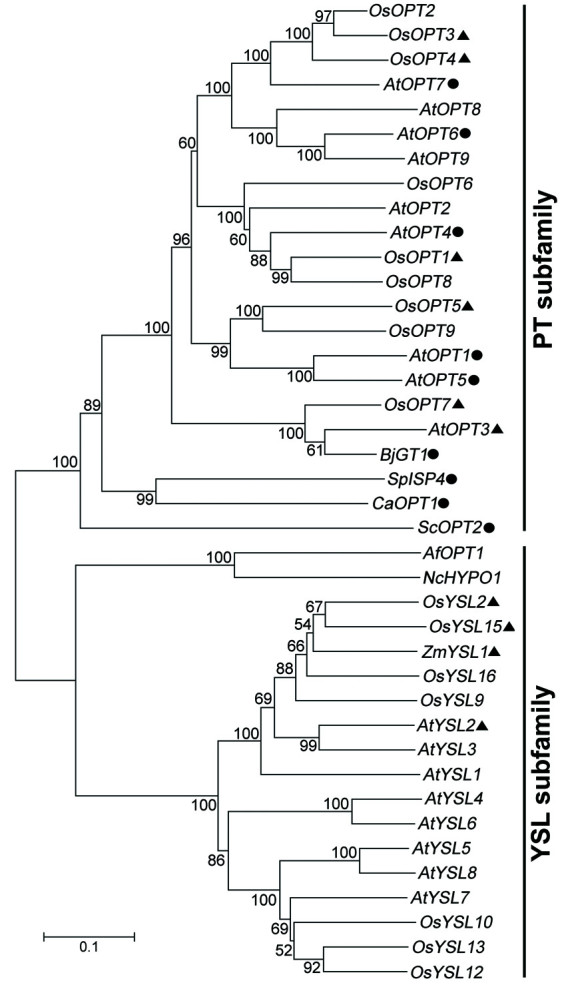
**Phylogenetic tree of the oligopeptide transporter (OPT) genes among rice and other species, based on amino acid sequences.** The tree was drawn according to results generated by MEGA4.0 analysis using the neighbor-joining method with an amino acid and Poisson correction model. Bootstrap values calculated for 1,000 replicates are indicated at corresponding nodes. The groups with the cycles were functionally demonstrated to transport oligopeptides and the groups with the triangles were experimentally demonstrated to transport metal compounds. Locus IDs of *OsOPT* genes from rice are given in Table 1, and gi numbers of OPTs from other species are given as: *AfOPT1*, gi|20145239|; *AtOPT1*, gi|9758213|; *AtOPT2*, gi|15218331|; *AtOPT3*, gi|18414644|; *AtOPT4*, gi|9759417|; *AtOPT5*, gi|4938497|; *AtOPT6*, gi|4469024|; *AtOPT7*, gi|3600039|; *AtOPT8*, gi|9759191|; *AtOPT9*, gi|9759190|; *AtYSL1*, gi|41352037|; *AtYSL2*, gi|41352039|; *AtYSL3*, gi|9759194|; *AtYSL4*, gi|41352041|; *AtYSL5*, gi|41352043|; *AtYSL6*, gi|41352045|; *AtYSL7*, gi|41352047|; *AtYSL8*, gi|41352049|; *BjGT1*, gi|30722286|; *NcHYPO1*, gi|9368956|; *ZmYSL1*, gi|10770865|; *CaOPT1*, gi|2367386|; *ScOPT2*, gi|6325452|; *SpISP4*, |19112445|.

**Figure 2:**
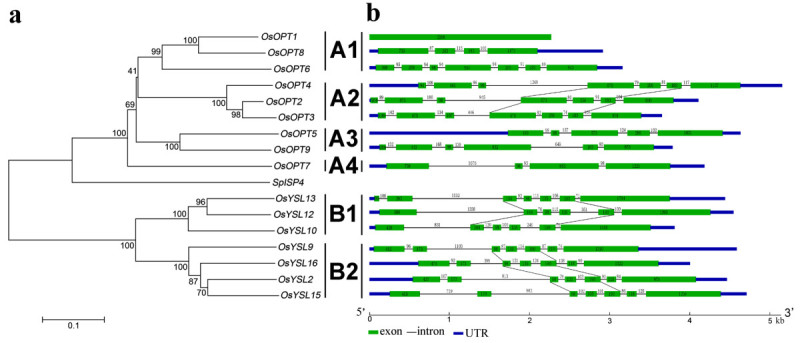
**Phylogenetic relationships (a) and gene structure schematic diagram (b) for** ***OsOPT*** **genes.** The phylogenetic tree was drawn as described in Figure 1 using *SpISP4* as an outgroup. The 16 *OsOPT* genes from rice were clustered into six sections. The identical exon formation of *OsOPT* genes is connected with slashes.

To further confirm the phylogenetic relationship of the *OPT* genes, we compared the rice *OsOPT* genes with *OPT* genes from other species. Some plant *OPT* genes had been experimentally demonstrated to have transportation functions for oligopeptides or metal chelates (Figure 1). These comparisons also suggested that the 16 genes could be classified into the same group as the OPT family homologues. By examining the phylogenetic relationship, nine of the 16 *OsOPT* genes were clustered into the PT subfamily and seven were clustered into the YSL subfamily (Lubkowitz [2006]; Yen et al. [2001]). Classification of the PT subfamily was in accordance with the peptide transport (PT) clade, which was identified and studied by (Lubkowitz [2006]). The nine *OsOPT* genes within the PT subfamily could be further classified into four sections (A1, A2, A3 and A4), and the seven genes within the YSL subfamily were further grouped into two sections (B1 and B2) (Figure 2a).

**Figure 3:**
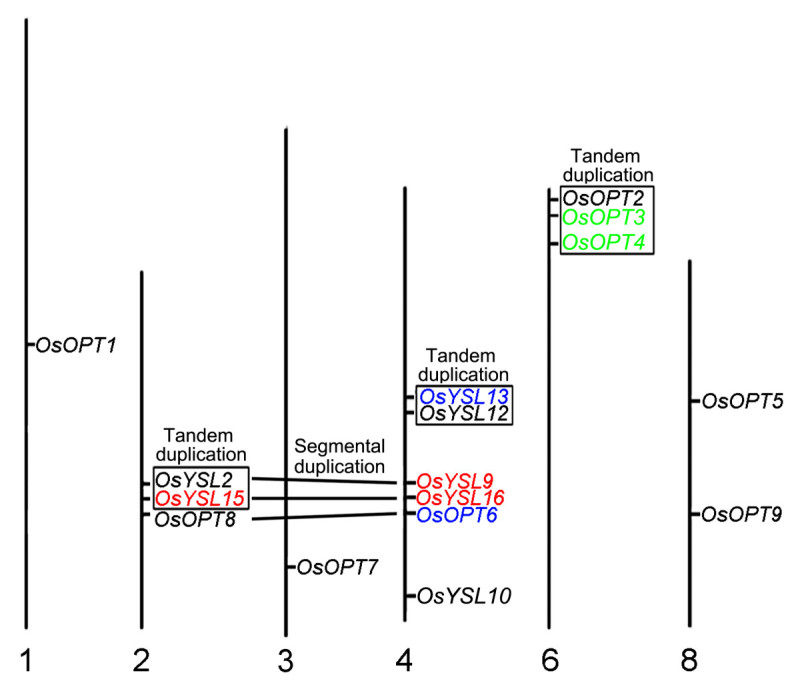
**Chromosomal location, duplication event, and evolutionary stage schematic for** ***OsOPT*** **genes.** Chromosome numbers are indicated at the bottom of each bar. The *OsOPT* genes represented by duplicated chromosomal segments between two chromosomes were connected by lines and the *OsOPT* genes that resulted from tandem duplication were contained in boxes. The *OsOPT* genes originating from the first evolutionary stage were shown in red, the *OsOPT* genes derived from the second evolutionary stage were shown in green, and the *OsOPT* genes derived from the third evolution stage were shown in blue.

The physical location of the 16 *OsOPT* genes could be assigned to the six rice chromosomes (Figure 3). Three genes found in section A1, *OsOPT1*, *OsOPT6* and *OsOPT8*, were localized to chromosome 1, 2 and 4, respectively. All three genes found in section A2 (*OsOPT2*, *OsOPT3* and *OsOPT4*) were localized to chromosome 6. Both *OsOPT5* and *OsOPT9* within section A3 were localized to chromosome 8. Section A4 only contained one gene, *OsOPT7*, which was localized to chromosome 3. All three genes found in section B1: *OsYSL10*, *OsYSL12* and *OsYSL13* were localized to chromosome 4. Among the four genes found in section B2, two genes (*OsYSL9* and O*sYSL16*) were localized to chromosome 4 and the other two (*OsYSL2* and *OsYSL15*) were localized to chromosome 2.

We also determined the intron-exon boundaries for the 16 *OsOPT* genes to obtain more information on the genomic organization of rice *OsOPT* genes (Figure 2b). According to the annotation of the rice genome, three of the six sections (A2, B1 and B2) had similar intron-exon boundaries. All three genes found in section A2 (*OsOPT2*, *OsOPT3* and *OsOPT4*) contain six introns and seven exons, but the length from the fourth to the sixth exon were identical. The genes in section B1 (*OsYSL10*, *OsYSL12* and *OsYSL13*) had four identical exons. All four genes found in section B2 (*OsYSL9*, *OsYSL16*, *OsYSL2* and *OsYSL15*) contain six introns and seven exons, but the length from the third to the fifth exon were identical. The identical exons were connected by slashes (Figure 2b). In addition, a 98 bp-exon was found in most of the 13 *OsOPT* genes except *OsOPT1*, *OsOPT3* and *OsOPT8*.

### Duplication and evolution analysis of the *OsOPT* genes

To reconstruct the expansion history of the 16 *OsOPT* genes in the rice genome, duplication and evolution analysis was performed. According to the duplication regions of rice chromosomes, we found that seven genes (*OsOPT2*, *OsOPT3* and *OsOPT4* in section A2, *OsYSL12* and *OsYSL13* in section B1, *OsYSL2* and *OsYSL15* in section B2) came from gene tandem duplication, which were shown in the box in Figure 3. However, three pairs (*OsYSL9*/*OsYSL16*, *OsYSL2*/*OsYSL15* and *OsOPT8*/*OsOPT6*) were located in duplicated chromosomal regions, suggesting that these six genes came from segmental duplication.

The nonsynonymous substitution rate (Ka), the synonymous substitution rate (Ks), and their ratio (Ka/Ks) are commonly used to aid in understanding the direction of evolution and its selective strength in a coding sequence (Li et al. [2009]). The Ka/Ks ratio is also a measure to explore the mechanism of gene divergence after duplication. Ka/Ks < 1 is termed “purifying selection”, which means natural selection eliminates deleterious mutations, thus maintaining the protein as is. Alternatively, a value where Ka/Ks > 1 indicates that selection has accelerated evolution by acting to change the protein, which is termed “positive selection”. Ka/Ks = 1 is referred to as “neutral selection”, meaning that natural selection does not have any forces acting on the protein (Hurst [2002]). To analyze the evolutionary selection after the duplication events of the five pairs of *OsOPT* genes, we calculated Ka, Ks and Ka/Ks ratios for the seven duplicated pairs in the OsOPT family (Table 2). The Ka/Ks ratio of five pairs (*OsYSL15/OsYSL2* *OsYSL16/OsYSL15* *OsOPT4/OsOPT3* *OsOPT4/OsOPT2* and *OsYSL9/OsYSL2*) was less than 1, suggesting that purifying selection was acting on these five duplicated pairs. These data indicated that the mutation of the duplicated OsOPT protein is much less likely to differ between the two homologues; that is, most of the time selection eliminates deleterious mutations. However, the Ka/Ks ratio of *OsYSL12/OsYSL13* and *OsOPT6/OsOPT8* were more than 1, suggesting that positive selection acts on the two duplicated pairs. These data provide strong evidence to suggest that selection between these two gene pairs has acted to change the OsOPT protein (positive selection).

**Table 2:** **Ka/Ks analysis and estimate of the absolute dates for the duplication events between the duplicated** ***OsOPT*** **genes**

Duplicated pair	Ka	Ks	Ka/Ks	Date (million years)	Duplicate type	Purifying selection
*OsYSL15*/*OsYSL2*	0.1546	0.9061	0.1706	69.70	Tandem	Yes
*OsYSL16*/*OsYSL15*	0.1679	0.8115	0.2069	62.42	Segmental	Yes
*OsYSL9*/*OsYSL2*	0.1955	0.9902	0.1974	76.16	Segmental	Yes
*OsYSL12*/*OsYSL13*	0.3304	0.1368	2.4152	10.52	Tandem	No
*OsOPT4*/*OsOPT3*	0.1139	0.6271	0.1816	48.23	Tandem	Yes
*OsOPT4*/*OsOPT2*	0.0791	0.5929	0.1334	45.60	Tandem	Yes
*OsOPT6*/*OsOPT8*	0.5302	0.2936	1.8058	22.58	Segmental	No

We also calculated the dates of the duplication events (Table 2) by molecular clock theory (Yu et al. [2005]) using the nucleotide substitution rate (Ks) and the neutral evolutionary rate (λ) of 6.5 × 10^−9^ substitutions per silent site per year for rice genome (Yu et al. [2005]). According to the first whole-genome duplication event of grass genomes (Gaut [2002]; Salse et al. [2008]), duplication events for the three pairs (*OsYSL15/OsYSL2, OsYSL16/OsYSL15* and *OsYSL9/OsYSL2*) occurred within the last 70 to 50 million years, after the origin of grasses (Gaut [2002]; Kellogg [2001]) and before the divergence of rice and maize (Gaut [2002]; Stebbins [1981]). Duplication events for the two pairs (*OsOPT4*/*OsOPT3* and *OsOPT4*/*OsOPT2*) occurred within the last 50 to 20 million years, after the divergence of rice and maize, but before *Zizaniinae* and *Oryzinae* were diverged from one another (Guo and Ge [2005]). Duplication events for the other two pairs (*OsYSL12*/*OsYSL13* and *OsOPT6*/*OsOPT8*) occurred within the last 20 to 9 million years, after *Zizaniinae* and *Oryzinae* were separated and before the *Oryza* genus branched off from the remaining genera of *Oryzeae* (Guo and Ge [2005]). Therefore, the evolutionary origin of the 16 *OsOPT* genes may have occurred in three evolutionary stages. The genes that came from the three evolutionary stages were shown in three different colors (Figure 3).

### Differential expression of the *OsOPT* genes under normal growth conditions

**Figure 4:**
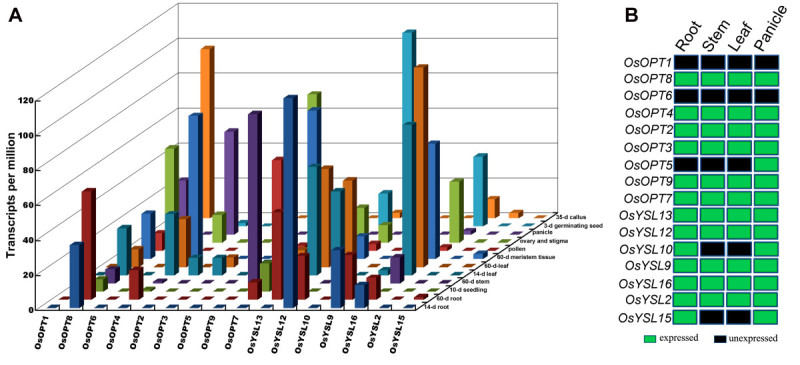
**Expression profiles of** ***OsOPT*** **genes in various rice organs. A.** Transcript abundance of *OsOPT* genes in tissue-specific libraries from the MPSS database. The transcript abundance of *OsOPT* genes were normalized to transcripts per million. **B.** Organ-specific expression analysis of *OsOPT* genes at booting stages by RT-PCR. The rice plants were grown in a normal, natural field.

Organ-specific and developmental expression of the 16 *OsOPT* genes under normal growth conditions were obtained from the rice MPSS database and RT-PCR (Figure 4). The data showed that expression profiles of the *OsOPT* genes were different during various developmental stages and in organs. The expression profiles for most *OsOPT* genes from the MPSS database (Figure 4A) matched well with the results from RT-PCR (Figure 4B). At the booting stage (Figure 4B), results from the RT-PCR showed that eleven of the 16 *OsOPT* genes were constitutively expressed in all four tested organs, three genes (*OsOPT5* *OsYSL10* and *OsYSL15*) were expressed in at least one organ and two genes (*OsOPT1* and *OsOPT6*) were not expressed. However, when comparing the expression of *OsOPT* genes from the Rice Expression Profile Database (RiceXPro: http://ricexpro.dna.affrc.go.jp/index.html) (Sato et al. [2010]), we found that *OsOPT6* was specifically expressed in the 1.2-2.0 mm portion of the anthers (data not shown). Therefore, we confirmed that genes were expressed under normal growth conditions except *OsOPT1*, which also did not have a corresponding mRNA sequence in GenBank (Table 1).

Data from the MPSS database suggested that some genes showed differential expression profiles in the developmental organs (Figure 4A). For the vegetative organs, four genes (*OsOPT4*, *OsOPT7*, *OsYSL13* and *OsYSL12*) were differentially expressed among the roots of 14-day-old seedlings and 60-day-old seedlings; however, three genes (*OsOPT4*, *OsOPT7*, *OsYSL13*) were expressed at a higher level and one gene (*OsYSL12*) was expressed at a lower level in the 60-day-old roots than the 14-day-old roots. Three genes (*OsOPT2*, *OsOPT7* and *OsOPT9*) had lower expression in the 14-day-old leaves than the 60-day-old leaves. For the reproductive organs, only five genes (*OsOPT5*, *OsOPT8*, *OsOPT9*, *OsOPT12* and *OsYSL16*) were expressed in the male organs (mature pollen); whereas, six genes (*OsOPT4*, *OsOPT8*, *OsOPT9*, *OsYSL12*, *OsYSL13* and *OsYSL16*) were expressed in the female organs (ovary and mature stigma). In addition, seven genes were expressed in the 35-day-old callus.

### Expression of the *OsOPT* genes under abiotic and biotic stresses

**Figure 5:**
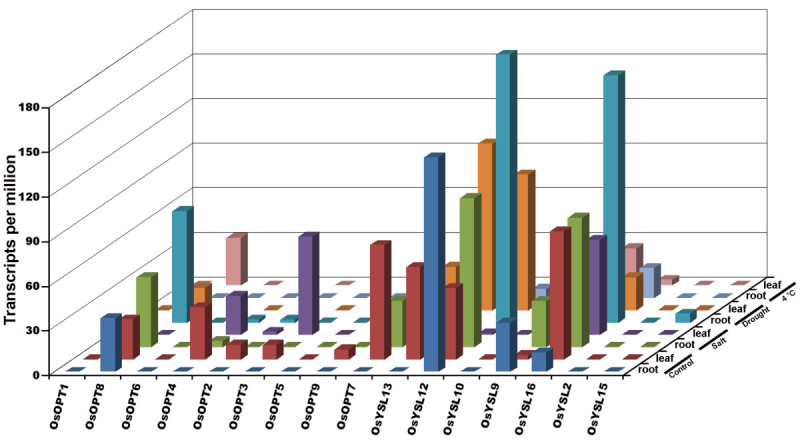
**Expression profiles of** ***OsOPT*** **genes under abiotic stress treatments.** The data were from the MPSS database. Roots and leaves from 14-d- seedlings stressed in 250 mM NaCl (salt) for 24 h, lack of water (drought) for 5d, and in 4 °C cold for 24 h. Roots and leaves from 14-d- seedlings were used as a control.

**Figure 6:**
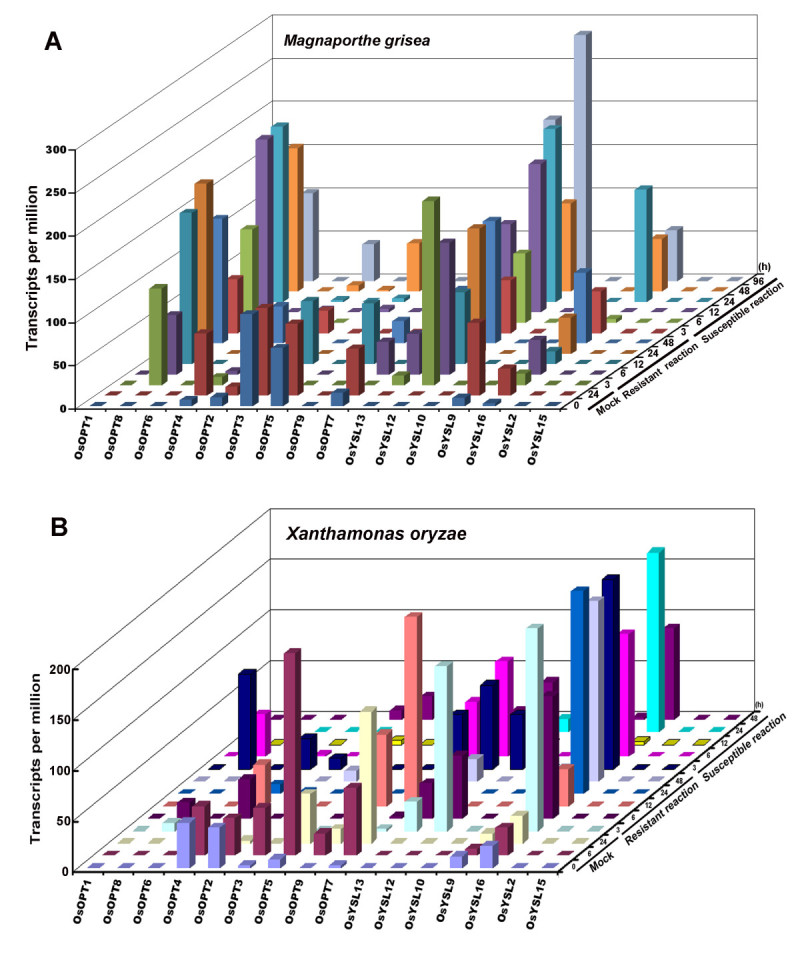
**Differential expression of** ***OsOPT*** **genes in response to** ***Magnaporthe grisea*** **, and** ***Xanthomonas oryzae.*** These data were from the MPSS database. **A.** Expression after *X. oryzae* inoculation. The resistant reaction group was leaves from a 2-month-old Nipponbare-Xa21 treated with *X. oryzae* (resistant variety) and the susceptible reaction group was leaves from a 2-month-old Nipponbare-Xa21 treated with *X. oryzae* (susceptible variety). The mock treatment group was leaves from a 2-month-old Nipponbare-Xa21 treated with water. **B.** Expression after *M. grisea* inoculation. The resistant reaction group was leaves from a 3-week-old Nipponbare-Pi9 treated with *M. grisea* (resistant variety) and the susceptible reaction group was leaves from a 3-week-old Nipponbare treated with *M. grisea* (susceptible variety). The mock treatment group was leaves from a 3-week-old Nipponbare treated with water.

To gain insight into the comprehensive roles of the OsOPT family members in response to various stresses, their expression patterns were investigated in rice seedlings subjected to abiotic (salt, drought and cold) (Figure 5) and biotic (two rice diseases) stresses (Figure 6) by mining the MPSS database. Only the genes, whose expression have at least a two-fold increase or decrease, were considered to have responded to stresses. Among the 16 *OsOPT* genes, nine genes showed differential expression in at least one tissue or at least one stress treatment compared to the control.

In response to salt (250 mM NaCl) treatment, four genes (*OsOPT4*, *OsOPT7*, *OsYSL13* and *OsYSL16*) were upregulated in the roots; however, five genes (*OsOPT2*, *OsOPT7*, *OsOPT9*, *OsYSL13* and *OsYSL12*) were downregulated and one gene (*OsOPT3*) was upregulated in the leaves. In response to drought treatment, seven genes (*OsOPT2*, *OsOPT4*, *OsOPT7, OsOPT8, OsYSL13 OsYSL15* and *OsYSL16*) were upregulated and one gene (*OsYSL9*) was downregulated in the roots; however, only two genes (*OsYSL12* and *OsYSL13*) were upregulated and six genes (*OsOPT2*, *OsOPT3*, *OsOPT4, OsOPT7*, *OsOPT9* and *OsYSL16*) were downregulated in the leaves. In response to cold (4^o^C) treatment, three genes (*OsOPT8*, *OsYSL9* and *OsYSL12*) in the roots and seven genes (*OsOPT2*, *OsOPT3*, *OsOPT4*, *OsOPT7, OsOPT9*, *OsYSL13* and *OsYSL16*) were downregulated in the leaves, and only one gene (*OsYSL9*) was upregulated in the leaves.

We also analyzed the responses of the *OsOPT* genes to infection with a fungus, *Magnaporthe grisea,* or a bacteria, *Xanthomonas oryzae* (Figure 6), which are known to cause rice blast and rice bacterial streak, two main rice diseases that cause severe decreases in rice yield. The expression changes of *OsPTRs* were determined after the inoculation. The microarray data and the infection with *M. grisea* and *X. oryzae* were published previously (Nobuta et al. [2007]). We found that four genes (*OsOPT8* *OsYSL12* *OsYSL13* and *OsYSL16*) were upregulated and three genes (*OsOPT3* *OsOPT5* and *OsYSL9*) were downregulated by *M. grisea* treatment (Figure 6A), and there was no response difference among these seven genes between the resistance reaction and the susceptible reaction of the rice variety Nipponbare. In response to *X. oryzae* treatment (Figure 6B), upregulated expression of two genes (*OsOPT8* and *OsYSL12*) were observed only during the early inoculation, and two genes (*OsYSL13* and *OsYSL16*) were upregulated during the whole inoculation; and two genes (*OsOPT2* and *OsOPT5*) showed downregulation.

### Expression response of the *OsOPT* genes to hormone treatments

**Figure 7:**
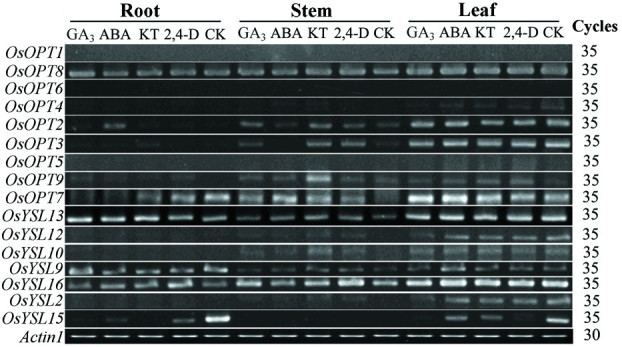
**Expression profiles of** ***OsOPT*** **genes under various hormone treatments.** Three-week-old seedlings were irrigated with Hoagland's Solution containing 5 μM gibberellic acid (GA_3_), 5 μM 2,4-dichlorophenoxyacetic acid (2,4-D), 5 μM kinetin (KT), or 25 μM abscisic acid (ABA) for 24 h. Control (CK) was from the water-treated seedlings. *Actin1* was used as an internal control.

We used semi-quantitative RT-PCR to monitor the effect various hormones would have on the expression of *OsOPT* genes. A total of fourteen genes were amplified in three-week-old rice seedlings subjected to various hormone treatments, including 2,4-dichlorophenoxyacetic acid (2,4-D, an auxin), kinetin (KT, a cytokinin), abscisic acid (ABA) and gibberellic acid (GA_3_) (Figure 7). The results indicated that the expression of most (11) *OsOPT* genes were not regulated by the four phytohormone treatments. However, the expression of *OsYSL15* was downregulated by the four hormones in the roots and the leaves, *OsOPT7* was dowregulated by GA_3_ and ABA in the roots, and *OsOPT2* was upregulated by ABA in the roots.

### Primary phenotypes of the inserted mutants for five *OsOPT* genes

To acquire more clues regarding the biological function of *OsOPT* genes, we searched main rice inserted mutant databases. Inserted mutants of all sixteen *OsOPT* genes were found in different mutant databases (Additional file 1: Table S1). However, the phenotype records of mutants of only five *OsOPT* genes were found in two mutant databases and their observed phenotypes were integrated into Table 1. The segregation rate and the plant numbers recorded in the mutant database can be found in Additional file 1: Table S1. From the records of the database, we found that some plants of the five *OsOPT* mutants displayed abnormal phenotypes under normal growth conditions (Table 1, Additional file 1: Table S1). Of course, it should be further investigated whether the phenotypes of the mutants from the database are linked with the five *OsOPT* genes.

**Figure 8:**
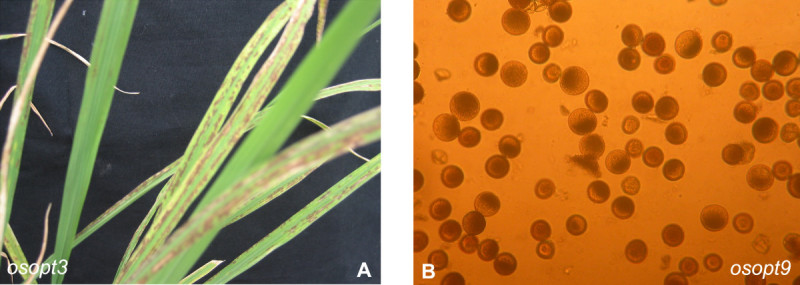
**Main phenotypes of two** ***OsOPT*** **inserted mutants (** ***osopt3*** **and** ***osopt9*** **). A.** Leaf lesions from a homozygote *osopt3* mutant. **B.** Semi-sterile pollens from a homozygote *osopt9* mutant. The homozygote plants (*osopt3* and *osopt9*) were grown in natural fields during normal rice season in Guangzhou, China.

From National Institute of Agrobiological Sciences (NIAS) of Japan (Miyao et al. [2003]), we obtained the *Tos17*-insertion mutants of *OsOPT3* (*OsGT1*) and *OsOPT9*. The inserted loci for *OsOPT3* (*OsGT1*) and *OsOPT9* were confirmed by flanking sequence method. Then the *osptr3* and *osptr9* homozygote mutants were identified from the offspring by PCR method and were use for phenotype observation. The homozygote *osptr3* mutant plant showed signs of serious lesion mimic symptom in the leaves even though they were grown under natural field (Figure 8A) and this symptom was not observed when the homozygote *osptr3* mutant plant was grown in shade (data not shown). In addition, the homozygote *osptr9* mutant mainly had semi-sterile pollen (Figure 8B) and low seed setting (data not shown). However, we did not observe the semi-dwarf and low tillering phenotype in the *osopt9* mutants, which were observed in the mutant database as shown in Table 1.

## Discussion

### Evolutionary expansion of the *OsOPT* gene family in rice

Gene duplication plays an important role in the expansion of a gene family (Adams and Wendel [2005]; Hughes [1994]). Several mechanisms have been suggested to drive the expansion of a gene family, including segmental duplication, tandem duplication and retroposition (Cannon et al. [2004]; Kong et al. [2007]). In this study, we identified sixteen members of the *OsOPT* gene family from the rice genome. We found that both the segmental duplication and the tandem duplication contributed to the evolutionary expansion of the *OsOPT* gene family in rice.

To speculate on the evolutionary orders of the rice OsOPT family, we considered protein sequence similarities with SpISP4 (Table 1), dates of the gene appearance (Table 2), rice genome evolutionary events (Krom and Ramakrishna [2010]), etc. The rice *OsOPT* members, which are more similar to *SpISP4* from *S. pombe*, might have evolved earlier in the rice genome (Krom and Ramakrishna [2010]). The molecular clock was also used to compute the date of duplication by calculating the number of substitutions per silent site (Ks). Yu et al. ([2005]) identified an evolutionary rate of 6.5 × 10^−9^ substitutions per silent site per year by analyzing the duplication history of the rice genome, dating the duplication event to 53 million years ago (Mya). Based on the dates of the duplication events (Table 2) and similar E-value to *SpISP4* (Table 1), we speculated that the evolutionary expansion of the *OsOPT* gene family in the rice genome underwent three stages. The three evolutionary stages of the *OsOPT* genes were shown with three colors in Figure 3. Before the evolutionary expansion of the *OsOPT* gene family in the rice genome, nine *OsOPT* genes (gene name in black) were distributed on six chromosomes (Figure 3). The first evolutionary stage was from the origin of the grasses (70 million years ago) to the divergence of rice and maize (approximately 50 million years ago). The original nine *OsOPT* gene homologues were also found in the maize genome (data not shown). During the first stage, *OsYSL15* arose from *OsYSL2* located on chromosome 2 after one tandem duplication event. Next, through one segmental duplication event between chromosome 2 and chromosome 4, *OsYSL9* and *OsYSL16* arose from *OsYSL2* and *OsYSL15* on chromosome 4. The four newly evolved genes were shown in red (Figure 3). The second evolutionary stage occurred at the point of divergence between rice and maize (approximately 50 million years ago) to the separation of *Zizaniinae* and *Oryzinae* (approximately 20 million years ago). In the second stage, *OsOPT4* and *OsOPT3* arose from *OsOPT2* on chromosome 6 by two tandem duplication events. The two newly evolved genes were shown in green (Figure 3). The third evolutionary stage was from the separation of *Zizaniinae* and *Oryzinae* (approximately 20 million years ago) to the point where *Oryza* branched-off from the remaining *Oryzeae* genera (approximately 9 million years ago). In the third stage, *OsYSL13* arose from *OsYSL12* on chromosome 4 after one tandem duplication event; *OsOPT6* arose from *OsOPT8* on chromosome 4 following one segmental duplication event. The two newly evolved genes were shown in blue (Figure 3). After the three evolutionary stages, the number of the rice *OsOPT* gene family increased to sixteen genes. Notably, purifying selection occurred at stage 1 and stage 2, but positive selection occurred at stage 3.

### Different functions and multiple roles of the *OsOPT* genes in rice development

Some plant *OPT* genes have been characterized in embryo development (Stacey et al. [2005]), glutathione transport (Zhang et al. [2004]), heavy metal detoxification (Cagnac et al. [2004]), seed germination (Muntz [1998]), and long distance nitrogen allocation (Williams and Miller [2001]). The genome-wide expression analysis of the *OsOPT* gene family showed that eleven genes (*OsOPT2* *OsOPT3* *OsOPT 4* *OsOPT7* *OsOPT8* *OsOPT9* *OsYSL2* *OsYSL9* *OsYSL12* *OsYSL13* and *OsYSL16*) had transcript accumulation in all tissues, and some in germinating seeds, roots, panicles and callus (Figure 4). These data suggested multiple functions for these oligopeptide transporters in rice and they might be involved in housekeeping functions during rice growth and development. On the other hand, some genes may play roles in specific tissues. The expression of *OsOPT6* was not detectable in most of the tested tissues (Figure 4), but it was specifically expressed in the 1.2-2.0 mm portion of the anthers (Data from RiceXpro expression database).

*AtOPT6* (Pike et al. [2009]; Cagnac et al. [2004]) and *OsOPT3* (*OsGT1*) (Zhang et al. [2004]) have been characterized GSH transporters and have been suspected of having a role in mediating heavy metal phytotoxicity. However, little is known about the role of the *OsOPT* genes in response to stress. We obtained some clues regarding the role of *OsOPT* genes in response to stresses by examining their expression level. Nine of the 16 *OsOPT* genes (*OsOPT2* *OsOPT4* *OsOPT7* *OsOPT7* *OsOPT9* *OsYSL12* *OsYSL13* *OsYSL15* and *OsYSL16)* showed a response to at least one abiotic stress treatment (Figure 5). Of the two main rice disease treatments, the expression of seven genes (*OsOPT3* *OsOPT5 OsOPT8*, and *OsYSL9* *OsYSL12* *OsYSL13* and *OsYSL16*) changed after *M. grisea* treatment and the expression of six genes (*OsOPT2* *OsOPT5* *OsOPT8* *OsYSL12* *OsYSL13* and *OsYSL16*) changed after *X. oryzae* treatment (Figure 6).

### Functional diversification of *OsOPT* genes

Functional diversification is a result of the evolutionary expansion of a gene family by gene duplication and often accompanies changes in the expression profile of the gene family member (Hughes [1994]; Lynch and Conery [2000]). The OPT family has been divided into two subfamilies (the PT and YSL subfamily) (Curie et al. [2001]), where the YSL subfamily was believed to be able to transport iron compounds and the PT subfamily was believed to be able to transport oligopeptides with four-five amino acid residues (Lubkowitz [2006]). However, several members of the PT subfamily have also been identified as transporters of iron compounds, such as *AtOPT3* (Stacey et al. [2007]), *OsOPT1* *OsOPT4* and *OsOPT7* (Vasconcelos et al. [2008]). These data may support the idea that the two subfamilies originated from a common ancestor.

However, expression diversification between the duplicated pairs could be found in all seven duplicated genes (Table 3), indicating that functional diversification occurred following the duplication event. Based on the *OsOPT* gene expression data from the MPSS database and our experimental results, we found some characteristics of the *OsOPT* gene expression profiling in this study, including the following. (1) The rice *OsOPT* genes were differentially expressed at various developmental stages and in tissues. Eleven genes (*OsOPT2*, *OsOPT3*, *OsOPT4*, *OsOPT7*, *OsOPT8*, *OsOPT9*, *OsYSL2*, *OsYSL9*, *OsYSL12*, *OsYSL13* and *OsYSL16*) showed transcript accumulation in all tissues (Figure 4B). However, subtle differences in the expression level of the eleven genes could be found in the developing plants; *OsOPT4*, *OsOPT7*, *OsYSL13* and *OsYSL12* were differentially expressed between the roots of 14-d -seedlings and 60-d -seedlings. (2) The expression profiles of the duplicated *OsOPT* genes had distinct tissue specificities and response to stimuli. (3) Fifteen *OsOPT* genes were expressed and the expression of one gene (*OsOPT1*) could not be confirmed.

**Table 3:** **Expression diversification between the duplicated** ***OsOPT*** **genes**

Duplicated pair	Tissues	Abiotic stress			Biotic stress	
		Salt	Drought	Cold	***X. oryzae***	***M grisea***
*OsYSL15*/*OsYSL2*	+	-	-	-	-	-
*OsYSL16*/*OsYSL15*	+	+	+	+	+	
*OsYSL9*/*OsYSL2*	+	+	+	+	+	-
*OsYSL12*/*OsYSL13*	+	+	+	+	+	+
*OsOPT4*/*OsOPT3*	+	+	+	-	+	+
*OsOPT4*/*OsOPT2*	+	+	-	-	+	+
*OsOPT6*/*OsOPT8*	+	+	+	+	+	+

+, Expression difference exists between the two members of the pair, -, No expression difference between the two members of the pair.

The transport functions and the expression patterns of the sixteen *OsOPT* genes have been diversified following their evolution. In the PT subfamily, three members (*OsOPT1* *OsOPT4*, and *OsOPT7*) have been shown to be able to transport iron-chelates in yeast (Vasconcelos et al. [2008]). *OsOPT3* (*OsGT1*) could also transport glutathione, some amino acids and peptides (Zhang et al. [2004]). In the YSL subfamily, *OsYSL2* (Koike et al. [2004]) and *OsYSL15* (Inoue et al. [2008]; Lee et al. [2009]) have been shown to be able to transport metal-nicotianamine. *OsOPT6* arose from *OsOPT8* by one segmental duplication event approximately 22.58 million years ago, but their expression profiles were different. *OsOPT8* was expressed in all developing organs (Figure 4) and it was also induced by *M. grisea*; however, *OsOPT6* was not expressed under normal growth conditions and during testing with biotic and abiotic stresses. *OsOPT6* is not a pseudogene because it has a corresponding full-length mRNA (AK070801) that was found in the RAD-DB database (Table 1) and it might be specifically expressed in the 1.2-2.0 mm portion of the anthers.

To our knowledge there are few biofunctional data about rice *OsOPT* genes. Some offspring of the five inserted *OsOPT* mutants showed abnormal symptoms which were recorded in the mutant database and the symptoms of the five *OsOPT* mutants were distinct (Table 1). Mutants of *OsYSL2* *OsYSL12* and *OsOPT3* were *Tos17* insertion and mutant of *OsOPT4* was T-DNA insertion. Of course, these phenotypes from the mutant database might not link with the *OsPTR* genes, because the mutant lines may carry multiple *Tos17* or T-DNA insertions and the tissue-culture induced mutations (Hirochika et al. [1996]). By observing the phenotypes of two homozygote T-DNA inserted mutant *osopt3* (*osgt1*) and *osopt9*, we found *osptr3* showed a lesion mimic phenotype when the *osptr3* mutant plant was grown under light of normal growth field (Figure 8A), but no this symptom under shade (data now shown). In addition, *OsOPT3* (*OsGT1*) has been found to be a glutathione transporter (Zhang et al. [2004]). Glutathione is the major endogenous antioxidant produced by the cells, participating directly in the neutralization of free radicals and reactive oxygen compounds (Szalai et al. [2009]). These suggested that *OsOPT3* (*OsGT1*) may play a role in tolerance to oxidative burst. The homozygote *osptr9* mutant showed semi-sterile pollens (Figure 8B), which suggested that *OsPTR9* may play a role in pollen development. These preliminary data could suggest that the rice OPT family may be necessary for rice growth and they may play diverse roles in rice.

The *OsOPT* gene family has been researched for more than ten years. In plants, it seems to have the ability to transport oligopeptide or metals, but due to the large number of potential substrates, the real roles of the *OsOPT* gene family are not fully understood. In this study, we described the evolutionary expansion and expression profiling of the rice *OsOPT* gene family and provided useful information for further study on the function of the *OsOPT* gene family in rice.

## Conclusions

Rice OPT family contains 16 genes, and is classified into the PT and YSL subfamily with 9 and 7 genes, respectively. The 16 *OsOPT* genes evolved from 9 original *OsOPT* genes after 3 evolutionary stages. Their expression patterns of 16 *OsOPT* genes and phenotypes of five *OsOPT* mutants are different.

## Methods

### Identification of *OsOPT* gene homologues

To identify putative *OPT* genes in the rice genome, a reference OPT protein (SpISP4) (GI:19112445) from *S. pombe* was chosen as a query sequence to search in the Rice Genome Annotation Project (http://rice.plantbiology.msu.edu/) by BLASTP. The protein sequences satisfied E < 0.1 were selected as the candidate proteins, which were compared with the *OPT* records in GenBank. Then, the physical and chemical parameters of the putative OsOPT proteins were computed by ProtParam in the ExPASy Proteomics Server (http://www.expasy.ch/tools/protparam.html). The prediction of transmembrane helices was performed by TMHMM Server v. 2.0 (http://genome.cbs.dtu.dk/services/TMHMM-2.0/). To confirm above *OsOPT* genes as the *OPT* gene homologue,we also analyzed the predicated OsOPT proteins in the Pfam database (http://www.sanger.ac.uk/Software/Pfam/search.shtml).

### Phylogenetic analysis and gene structure

Multiple sequence alignments of amino acid sequences were performed using ClustalX (version 2.0.9) (Thompson et al. [1997]). Unrooted phylogenetic and molecular evolutionary analyses were constructed using MEGA4.0 by the neighbor-joining method with a Poisson correction model, using 1000 replicates for bootstrap analysis (Tamura et al. [2007]). Exon-intron organization was determined using the genome browser in RAP-DB (http://rapdb.dna.affrc.go.jp/). Gene structures were displayed using Gene Structure Display Server (GSDS) (http://gsds.cbi.pku.edu.cn/chinese.php).

### Chromosomal localization and gene duplication

The chromosomal localization of the *OsOPT* genes was determined by mapview in NCBI (http://www.ncbi.nlm.nih.gov/projects/mapview/map_search.cgi?taxid=4530). The *OsOPT* genes present on the duplicated chromosomal segments were identified by segmental genome duplication of rice available at MSU-RGA (http://rice.plantbiology.msu.edu/analyses_search_blast.shtml) with the maximum distance permitted between collinear gene pairs of 100 kb. The *OsOPT* genes separated by a maximum of twenty genes were identified as tandem duplicated genes (Lynch and Conery [2000]; Moore and Purugganan [2005]).

### Ka/Ks analysis and calculation of the date of duplication events

The number of nonsynonymous substitutions per nonsynonymous site (Ka) and the number of synonymous substitutions per synonymous site (Ks) of duplicated genes were calculated with DnaSP Version 5. The duplication date could be computed with the number of substitutions per silent site (Ks) (Comeron [1999], Shiu et al. [2004]; Yang et al. [2008]). Yu et al. ([2005]) calculated a neutral evolutionary rate (λ) of 6.5 × 10^−9^ substitutions per silent site per year to analyze the duplication history of the rice genome. Here, we calculated the dates of the duplication events by the equation T = Ks/2λ, for rice, the λ = 6.5 × 10^−9^ (Yang et al. [2008]).

### Rice materials and treatments

The rice cultivar Nipponbare (*O. sativa* L. *japonica*) was used in this study. For expression analyzes of the *OsOPT* genes under normal conditions, total RNA was extracted from different tissues of the rice plants grown in the natural field of South China Botanical Garden, at the booting stage.

For phytohormone treatments, seeds were soaked in water and germinated at 28 °C for 2 days and then grown in Hoagland's Solution (Baba and Takahashi [1956]) under natural conditions for 3 weeks. Next, the seedlings were cultured in Hoagland's solution containing 5 μM gibberellic acid (GA_3_), 5 μM 2,4-dichlorophenoxyacetic acid (2,4-D, an auxin), 5 μM kinetin (KT, a cytokinin), or 25 μM abscisic acid (ABA), respectively, for 24 h.

For various treatments, published data were used as a reference for the growth conditions, treatments and experiments on the plant materials in the digital expression database (Nobuta et al. [2007]).

### RNA extraction and semi-quantitative RT-PCR analysis

Total RNA was extracted using Trizol reagent according to the manufacturer’s instructions(Invitrogen, http://www.invitrogen.com). First-strand cDNA was synthesized from 3 μg of total RNA treated with DNase I using M-MLV reverse transcriptase (Promega, http//http://www.promega.com). The first-strand cDNA was used as a template for semi-quantitative PCR after normalizing to rice *Actin1* (AB047313). Semi-quantitative RT-PCR was performed in a 20 μL reaction volume containing 1 μL cDNA solution, 1 × PCR buffer, 0.25 μM dNTPs, 1.0 μM gene-specific primers and 0.5 U Taq polymerase (Takara, Japan) using the following conditions: 94 °C for 3 min (1 cycle), 94 °C for 30 s, 60 °C for 30 s, 72 °C for 2 min (30-35 cycles) and 72 °C for 10 min (1 cycle). All primer sequences used in this study are listed in Additional file 2: Table S2. To ensure the primers were specific for the correct *OsOPT* genes, primers were designed using software Primer Primier v5.00 (PREMIER Biosoft international). The amplification products obtained with the different primer pairs were purified using gel electrophoresis and sequenced. Sequences were then aligned on the RAP-DB website to determine their specificity.

### Digital expression analysis using the MPSS database

The massively parallel signature sequencing (MPSS) database (http://mpss.udel.edu/rice/) was searched for 20-base signatures from 22 mRNA libraries representing 18 different tissues/organs in rice expression evidence analysis (Nakano et al. [2006]). Only the signatures uniquely identified for an individual gene and had a perfect match (100 % identity over 100 % of the length of the tag) were used for analysis. The normalized abundance (tags per million, tpm) of these signatures for a given gene in a given library represents the quantitative estimate of the expression of that gene. The 22 mRNA libraries of MPSS have been group into four groups to analyze the expression profile of the OsOPT family in various developmental stages, organs and various stress treatments.

### Biofunctional analysis of *OsOPTs*

Two approaches were used to obtain biofunctional clues on *OsOPTs*. First, different rice inserted mutant databases were searched. The information on the inserted mutants of *OsOPTs* was retrieved from OryGenesDB (an interactive tool for rice reverse genetics, http://orygenesdb.cirad.fr/index.html), searched by their gene locus ID, and the results were listed in Additional file 1: Table S1. Then, two mutant databases (OTL: http://urgi.versailles.inra.fr/OryzaTagLine/cgi-bin/focus_mutant.pl and NIAS: http://tos.nias.affrc.go.jp/index.html.en) were further searched using the plant names of the inserted mutants in Additional file 1: Table S1 to obtain the observed phenotypes of these mutants. Five *OsOPTs* mutants had their observed phenotypes in two mutant databases. The second approach was analysis of two *OsOPTs* (*OsOPT3* and *OsOPT9*) inserted mutants, which were from NIAS, and their main phenotypes were obtained from the homozygote *osopt3* and *osopt9* mutant plants.

## Electronic supplementary material

Additional file 1: **Table S1.** Information of the inserted mutants of OsOPTs in different mutant database. (DOC 46 KB)

Additional file 2: **Table S2.** List of the semi-quantitative RT-PCR primers used in this study. (DOC 187 KB)

Below are the links to the authors’ original submitted files for images.

Authors’ original file for figure 1

Authors’ original file for figure 2

Authors’ original file for figure 3

Authors’ original file for figure 4

Authors’ original file for figure 5

Authors’ original file for figure 6

Authors’ original file for figure 7

Authors’ original file for figure 8
