# Supplementary material for: Evolutionary expansion and functional diversification of oligopeptide transporter gene family in rice
Source: Rice (N Y). 2012 Jun 22;5:12. doi: 10.1186/1939-8433-5-12 (PMC5520842; doi:10.1186/1939-8433-5-12)
Supplement: Supplementary file 1 — Additional file 1: Table S1. Information of the inserted mutants of OsOPTs in different mutant database. (DOC 46 KB) [file 12284_2012_10_MOESM1_ESM.doc]

**Supplemental Table 1**: List of the semi-quantitative RT-PCR primers used in this study

| **Gene**  **name** | **Forward primer (5’-3’)** | **Reverse primer (5’-3’)** | **RT-PCR**  **cycles** | **Size of**  **RT-PCR products (bp)** |
| --- | --- | --- | --- | --- |
| *OsOPT1* | AAGGAGGAGAATAAGGAGGGC | GCAGAGCAAGAGCGAGACG | 35 | 711 |
| *OsYSL2* | AGAAAGCGACAGATGAGGA | AGCAGGGAAGAAGGGAAC | 35 | 880 |
| *OsYSL15* | GGGCTCCTCCTGGCTATT | GGTGAGATGTCCCGTCTTG | 35 | 996 |
| *OsOPT8* | CTCATCTCGCCCTTCTTT | TAGCCATACACCTTGAAACA | 35 | 662 |
| *OsOPT7* | TCATCGTGCCGCTCTGCT | CGGTTGCTGGTTGGTGGTG | 35 | 524 |
| *OsYSL13* | TGCTGTTGTTGCTGCCATTT | TCGCCCTGTCCACTCGTT | 35 | 704 |
| *OsYSL12* | CCTTCAGCGGTGGGTTTG | ATCGGGCTGCCATTGTTG | 35 | 733 |
| *OsYSL9* | TGATTTGCTCCCACCTTG | CCCATCTCCCATACTTCG | 35 | 942 |
| *OsYSL16* | CTGCTACACCATCGGCTACG | CGTCCGCCACTCTGTTGTTA | 35 | 746 |
| *OsYSL10* | TGGAAGGAGCAGGTGACGC | CGGAGATGTTGACGAGGTAAGG | 35 | 733 |
| *OsOPT6* | AGGTGTTGGGCTACGGATGG | CGAAGAAGAGGCTGAGGTGGA | 35 | 594 |
| *OsOPT2* | GGTTCCTGAGTGGTGGTT | TATTCTGCGTGGGCTAAT | 35 | 538 |
| *OsOPT3* | CATCTCCCTCCTTGTCGT | TGTTGCTTTGATTATCCCTA | 35 | 955 |
| *OsOPT4* | CTGTTGCGATCCTCAATG | AAGAACTGGGTGCGTGTC | 35 | 620 |
| *OsOPT5* | AGTTGCTTGGGTATGGATGG | CATGTTGGTCGTGGCAGAG | 35 | 953 |
| *OsOPT9* | CGATATGGCGGCAGACCA | CCAGATGATGGAGGCGTTGA | 35 | 596 |
| *Actin1* | GGACTCTGGTGATGGTGTCAGCCA | GAGCTGGTCTTGGCAGTCTCCA | 30 | 243 |
