# Supplementary material for: Evolutionary expansion and functional diversification of oligopeptide transporter gene family in rice
Source: Rice (N Y). 2012 Jun 22;5:12. doi: 10.1186/1939-8433-5-12 (PMC5520842; doi:10.1186/1939-8433-5-12)
Supplement: Supplementary file 2 — Additional file 2: Table S2. List of the semi-quantitative RT-PCR primers used in this study. (DOC 187 KB) [file 12284_2012_10_MOESM2_ESM.doc]

**Supplemental Table 1**: Information of the inserted mutants of *OsOPTs* in different mutant database

| **Genes** | **FST** | **Plant Name** | **Inserted region** | **Source^a^** | **Phenotypes of the mutant^b^** |
| --- | --- | --- | --- | --- | --- |
| *OsOPT1* | CZ167588 | RDs18_5.1 | exon | UCD |  |
| *OsYSL2* | AG023661 | ND1068_0_103_1A | 3’-UTR | NIAS | Dwarf, Albino, Fadded green leaf (1/ 25), Chlorina (1/ 25), Pale green leaf, Yellow, Drooping leaf( 1/ 18), Drooping leaf, Low tillering, Narrow leaf, White and yellow stripe( 1/ 18), Long clum( 5/ 18), Long culm, Semi Dwarf, Late heading |
|  | 3A-00607.L | A09257 | 3’-UTR | Postech |  |
|  | CZ168556 | RGT344_5.1 | exon | UCD |  |
| *OsYSL15* | 2D-10712.R | D03372 | promoter | Postech |  |
|  | 3A-10357.L | A22530 | exon | Postech |  |
|  | 3A-10357.L | A22531 | exon | Postech |  |
|  | 04Z11BI76 |  | exon | RMD |  |
|  | 04Z11BI76-2 |  | exon | RMD |  |
|  | EI007763 | M0045824 | promoter | TRIM |  |
| *OsOPT8* | K-00390.R | E00734 | promoter | Postech |  |
|  | 1A-22203.L | C03531 | exon | Postech |  |
|  | 1B-19707.L | C09688 | promoter | Postech |  |
|  | 2C-10489.L | C12297 | exon | Postech |  |
|  | 1B-03706.R | A02285 | promoter | Postech |  |
|  | 3A-11558.R | A24232 | exon | Postech |  |
|  | 04Z11KR88 |  | promoter | RMD |  |
|  | 05Z11CA15 |  | exon | RMD |  |
|  | 04Z11HA34 |  | promoter | RMD |  |
|  | CZ557042 | M0032980 | promoter | TRIM |  |
| *OsOPT7* | 1E-06012.R | C11069 | 3’-UTR | Postech |  |
|  | 1E-06018.R | C11071 | 3’-UTR | Postech |  |
|  | 3D-02585.R | D13365 | intron | Postech |  |
|  | 1E-06012.L | C11070 | 3’-UTR | Postech |  |
|  | 3A-18192.R | A33874 | intron | Postech |  |
| *OsYSL13* | 4A-50620.L | A46201 | intron | Postech |  |
|  | 04Z11MA04 |  | exon | RMD |  |
| *OsYSL12* | AG209522 | NE7024_0_401_1A | intron | NIAS | Sterile |
|  | AG207958 | NE1003_0_105_1A | exon | NIAS | Spl/Lesion mimic, The mutants whose the second leaf was browned were segregated in self-progenies (3/19) (for about 1 month seedling), Germination rate (25/29), Deep green leaf, Semi Dwarf (6/18), Withering, Low tillering (5/18), Wide leaf, Withering, Short panicle(5/18), |
|  | CL521951 | AKPA11 | promoter | OTL | Wide and long leaves |
|  | CL521950 | AKPH10 | promoter | OTL | Semi-dwarf (-15%) in proportion; semi-rolled and horizontal leaves |
|  | 1D-01542.R | B08852 | promoter | Postech |  |
|  | 2D-41364.R | D09653 | exon | Postech |  |
|  | 3A-15607.L | A30273 | intron | Postech |  |
|  | 3A-50541.L | A34736 | promoter | Postech |  |
|  | 1B-14738.L | C07655 | promoter | Postech |  |
|  | 2D-10826.L | D03522 | exon | Postech |  |
|  | 3A-06445.R | A16929 | exon | Postech |  |
|  | 3A-15607.R | A30271 | intron | Postech |  |
|  | 3A-15607.R | A30272 | intron | Postech |  |
|  | 3A-50541.R | A34737 | promoter | Postech |  |
|  | CZ557114 | M0033318 | exon | TRIM |  |
| *OsYSL9* | K-00606.R | E01199 | promoter | Postech |  |
|  | K-00606.L | E01200 | promoter | Postech |  |
|  | 3A-08505.R | A19861 | intron | Postech |  |
|  | EI009118 | M0059988 | intron | TRIM |  |
| *OsYSL16* | AY873061 | 04ET3_387_d_040_000_017H03 | exon | OSTID |  |
|  | 2D-10366.L | D02884 | intron | Postech |  |
|  | 1A-13015.R | A00620 | 5’-UTR | Postech |  |
|  | 05Z11CP34 |  | intron | RMD |  |
|  | 04Z11AN66 |  | exon | RMD |  |
|  | CZ552546 | M0003560 | promoter | TRIM |  |
|  | CZ552545 | M0003558 | promoter | TRIM |  |
| *OsOPT6* | CU314015 | AVWE12 | intron | OTL |  |
|  | 4A-01377.L | A41535 | intron | Postech |  |
|  | FI132042 | RGT6334_5.1 | intron | UCD |  |
| *OsYSL10* | 2D-30906.R | D07038 | promoter | Postech |  |
|  | 3D-50320.R | D14456 | promoter | Postech |  |
|  | 3A-15217.L | A29692 | promoter | Postech |  |
|  | 3A-15217.R | A29689 | 5’-UTR | Postech |  |
|  | 3A-15217.R | A29690 | 5’-UTR | Postech |  |
|  | CZ556838 | M0032213 | intron | TRIM |  |
|  | CZ556748 | M0031931 | exon | TRIM |  |
|  | DX576527 | RGT4595_5.1 | exon | UCD |  |
|  | DU133541 | RGT2127_5.1 | exon | UCD |  |
|  | FI132000 | RGT6371_5.1 | exon | UCD |  |
|  | FI131958 | RGT6339C_5.1 | 3’-UTR | UCD |  |
| *OsOPT2* | AY872832 | 01ET3_013_002_B_030_022G04 | promoter | OSTID |  |
|  | CU322758 | AKVE09 | 3’-UTR | OTL |  |
|  | 4A-01123.L | A41224 | promoter | Postech |  |
| *OsOPT3* | AG023377 | ND0043_0_104_1A | intron | NIAS | Strong green leaf (1/ 20), Deep green leaf, Glabrous leaf (9/ 20), Semi-short clum (2/ 20), Semi Dwarf |
|  | AG023558 | ND1017_0_401_1A | exon | NIAS | Spl/Lesion mimic, rolled leaf (5/ 20), Leaf tip withering (2/ 20), Withering, Very short clum (1/ 20), Extremely dwarf, White belly rice kernel |
|  | CU323410 | AKBC03 | exon | OTL |  |
|  | 4A-01123.R | A41225 | intron | Postech |  |
| *OsOPT4* | GS890792 | ASXE08 | exon | OTL | Yellow-green-1 Yellow-green plants with necrotic regions. Chlorosis. From yellow-green and/or yellow-reddish and/or pale green to totally yellow plants; delayed flowering; normal to small and weak plant; decreased plant size or semi-dwarf; smaller than average in line; low tillering; weak tillers. |
|  | GS890793 | ASXE08 | exon | OTL |  |
|  | CU313963 | AVWG01 | intron | OTL |  |
|  | ZSF6447 | ZSF6447 | 5’-UTR | SHIP |  |
|  | CZ553781 | M0015348 | exon | TRIM |  |
|  | EI007434 | M0040059 | 3’-UTR | TRIM |  |
|  |  | SHIP_ZSF6447 |  | SHIP |  |
| *OsOPT5* | 1A-17310.R | B00148 | promoter | Postech |  |
|  | ET025654 | RdSpm3667_3.1 | exon | UCD |  |
| *OsOPT9* | AB157195 | NG0095 | exon | NIAS | Semi Dwarf, Low tillering, Semi-sterile pollen |

^a^ The inserted mutants of *OsOPTs* were searched from OryGenesDB (<http://orygenesdb.cirad.fr/index.html>) by their gene locus ID. ^b^ The data were from the corresponding mutant database.
