# Supplementary figures and images for: Evolutionary expansion and functional diversification of oligopeptide transporter gene family in rice
Source: Rice (N Y). 2012 Jun 22;5:12. doi: 10.1186/1939-8433-5-12 (PMC5520842; doi:10.1186/1939-8433-5-12)

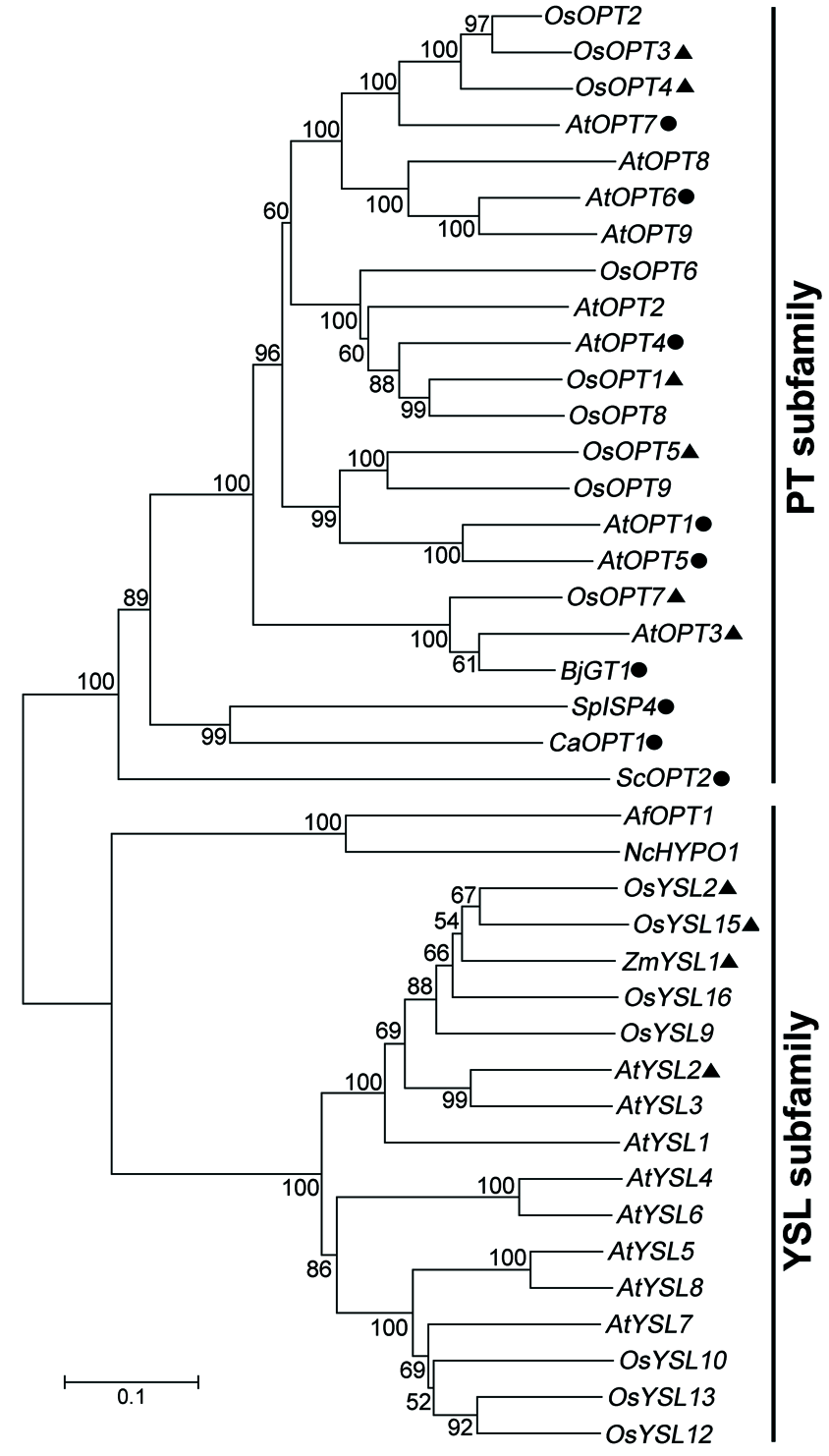

Supplement: Supplementary file 3 — Authors’ original file for figure 1 [file 12284_2012_10_MOESM3_ESM.tiff]

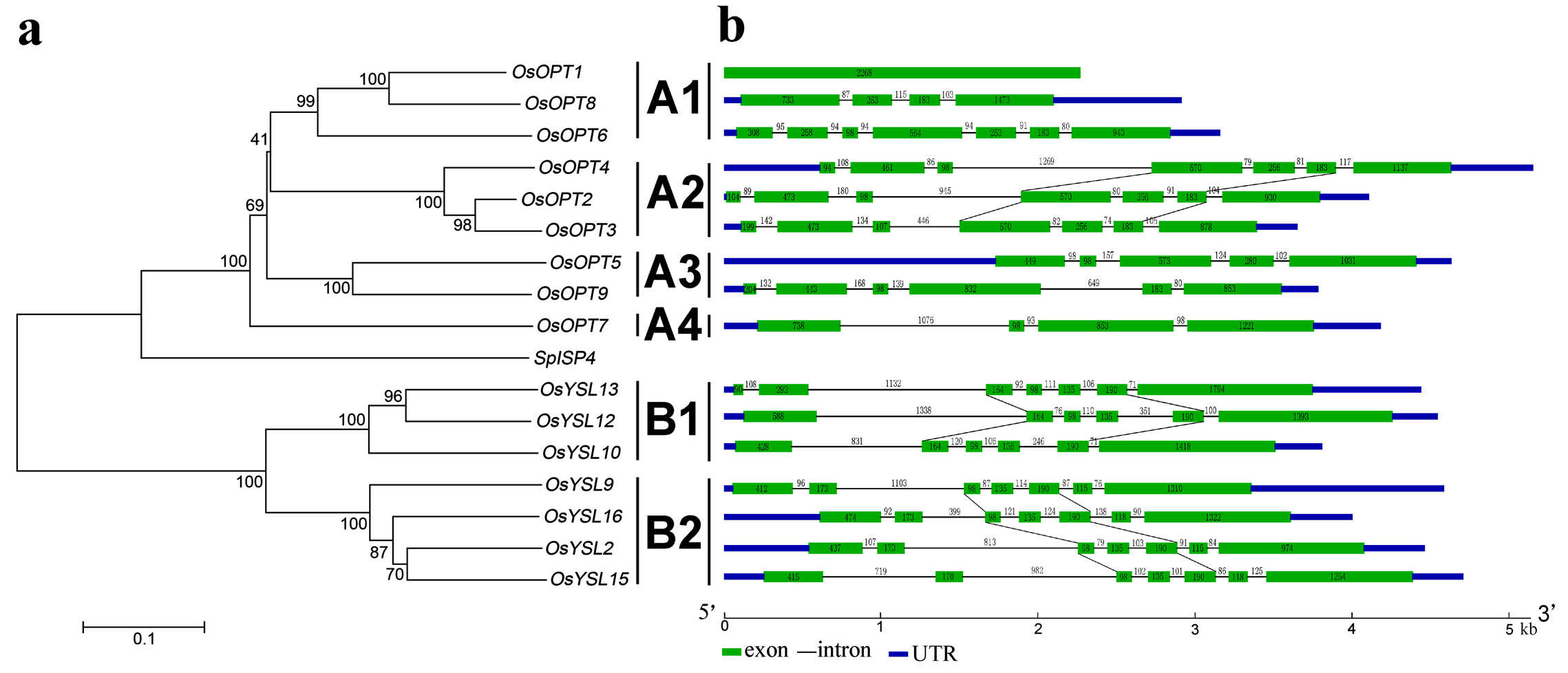

Supplement: Supplementary file 4 — Authors’ original file for figure 2 [file 12284_2012_10_MOESM4_ESM.tiff]

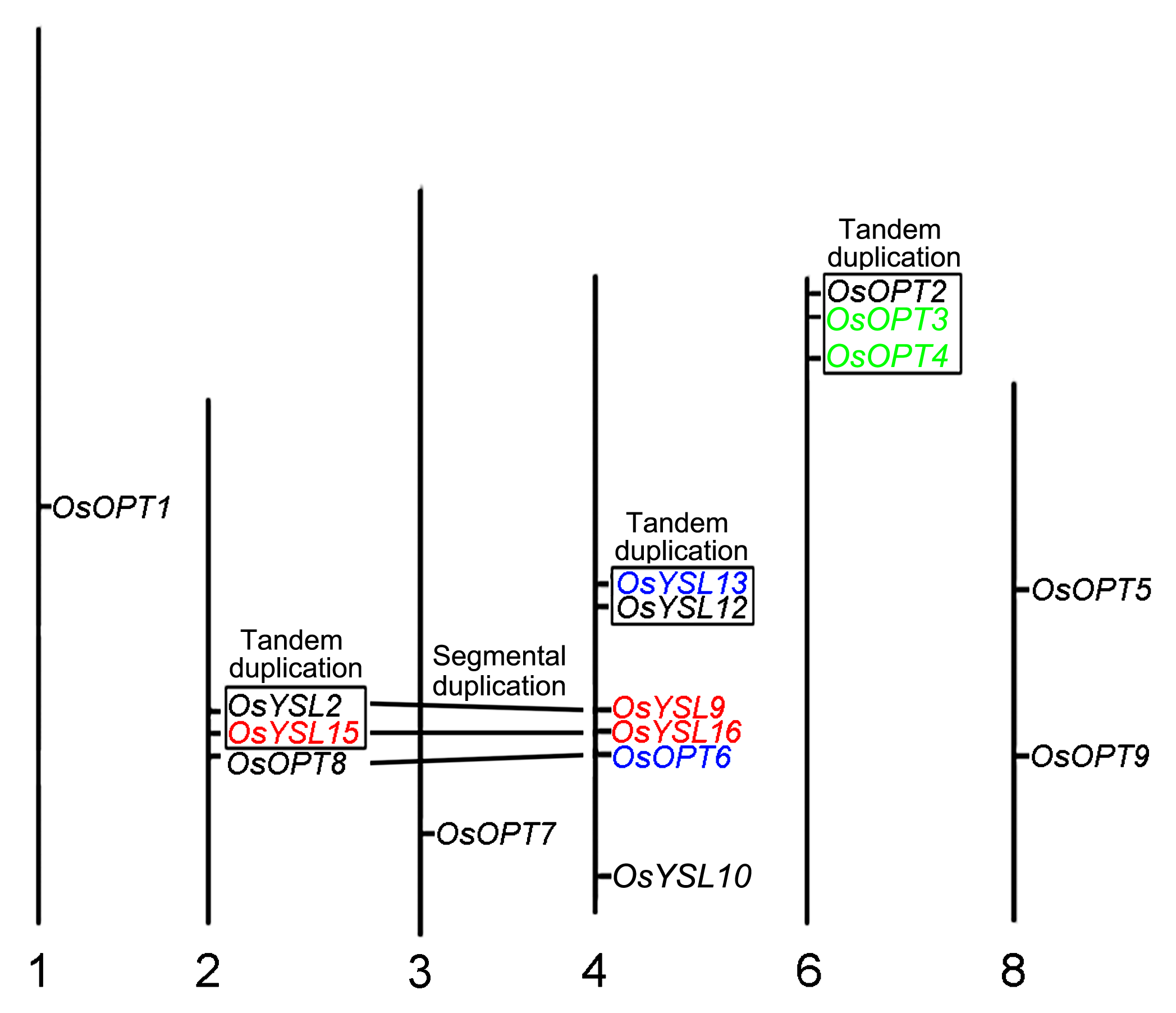

Supplement: Supplementary file 5 — Authors’ original file for figure 3 [file 12284_2012_10_MOESM5_ESM.tiff]

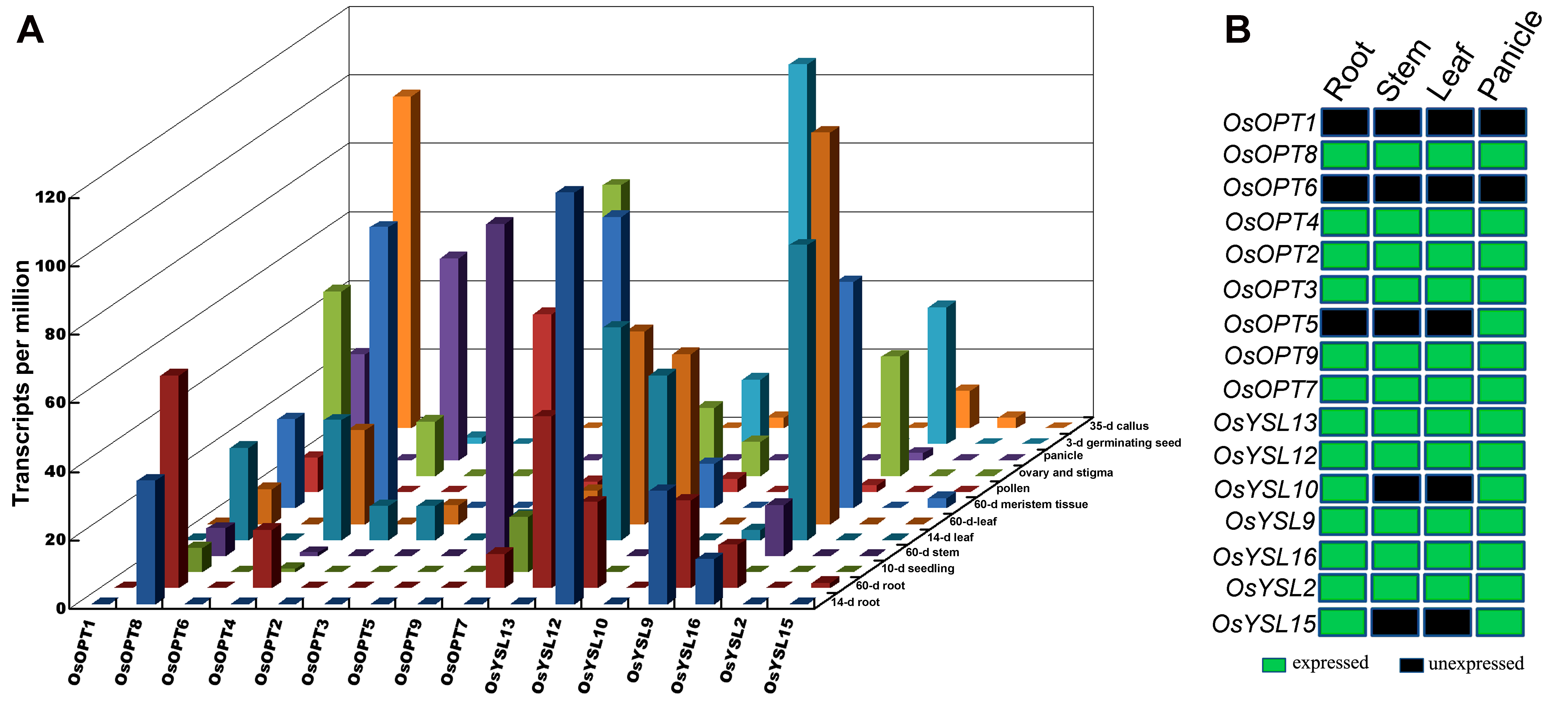

Supplement: Supplementary file 6 — Authors’ original file for figure 4 [file 12284_2012_10_MOESM6_ESM.tiff]

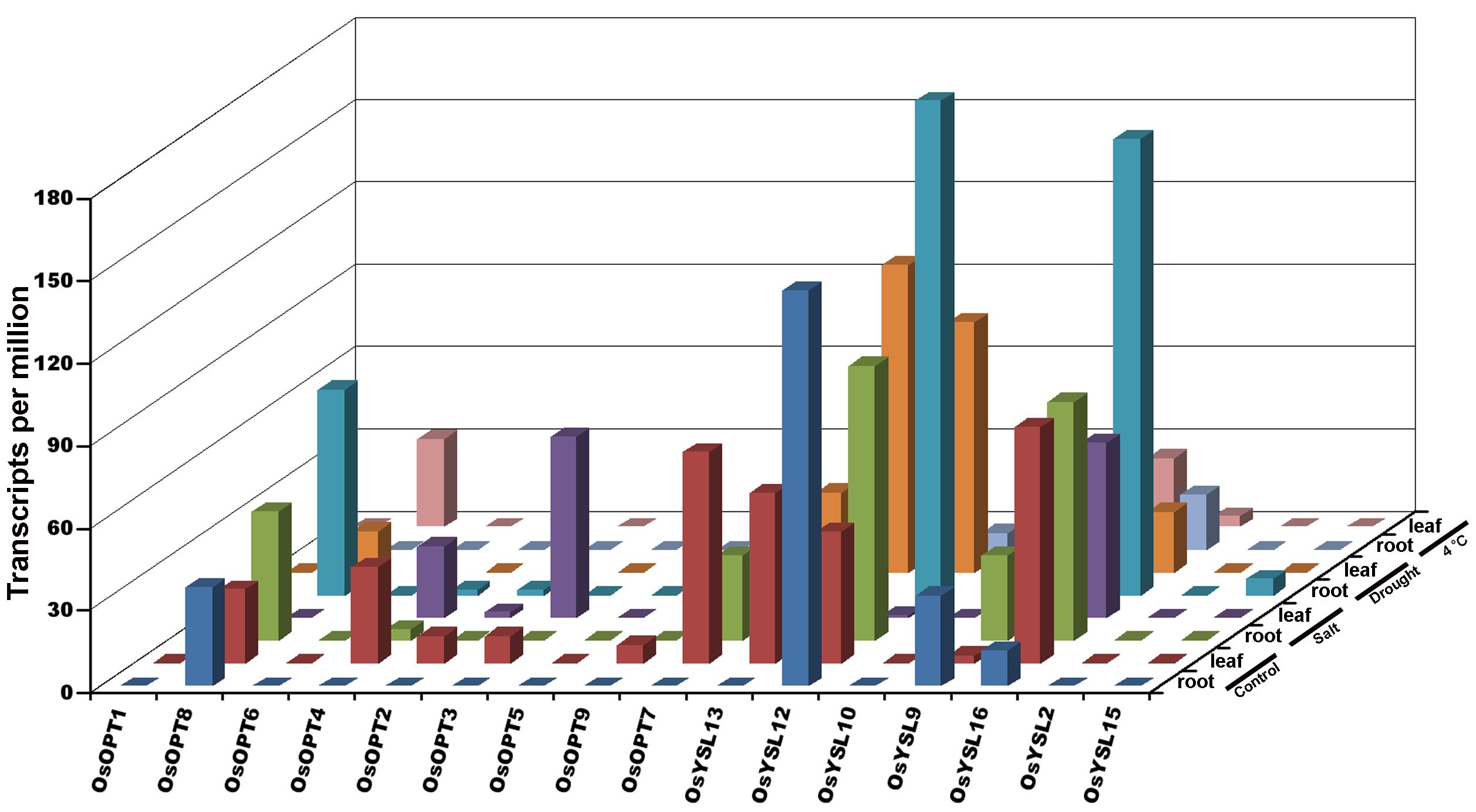

Supplement: Supplementary file 7 — Authors’ original file for figure 5 [file 12284_2012_10_MOESM7_ESM.tiff]

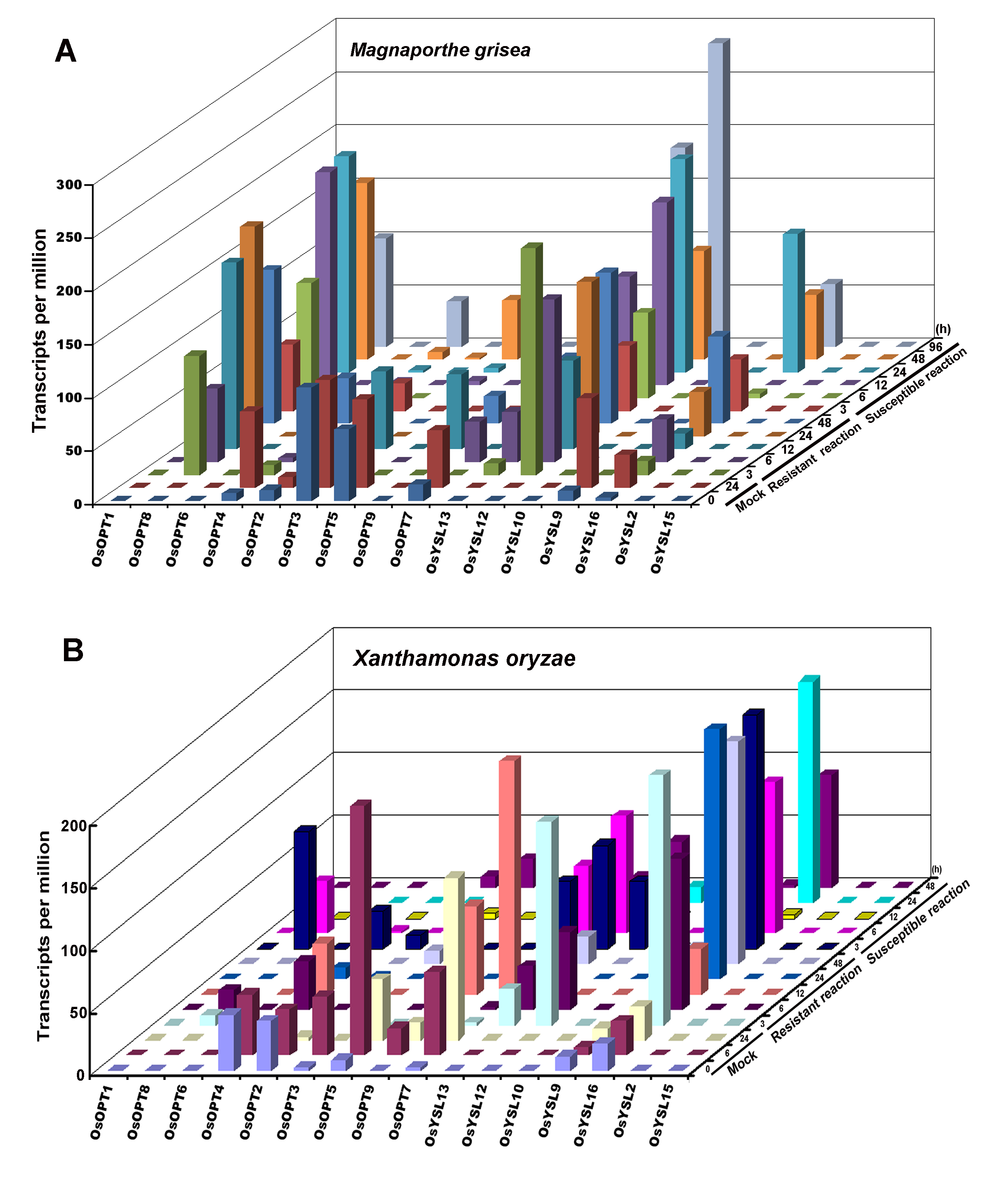

Supplement: Supplementary file 8 — Authors’ original file for figure 6 [file 12284_2012_10_MOESM8_ESM.tiff]

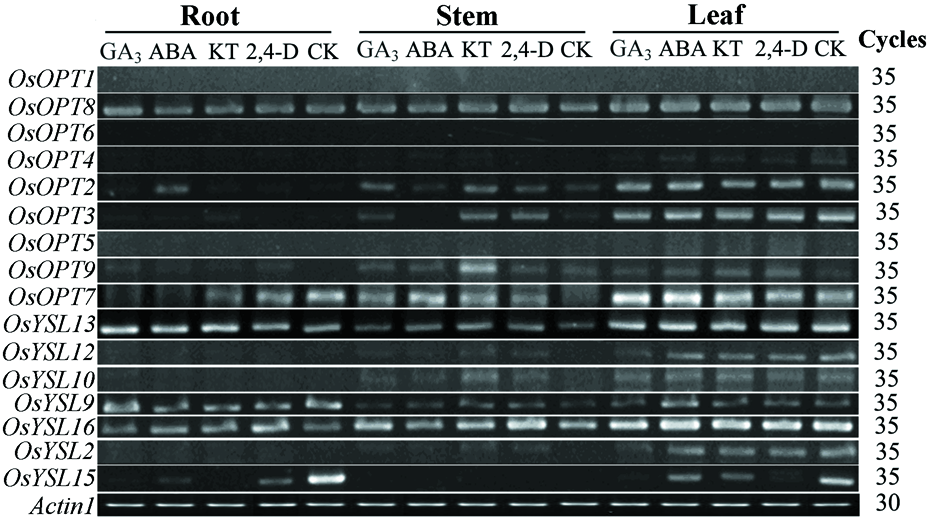

Supplement: Supplementary file 9 — Authors’ original file for figure 7 [file 12284_2012_10_MOESM9_ESM.tiff]

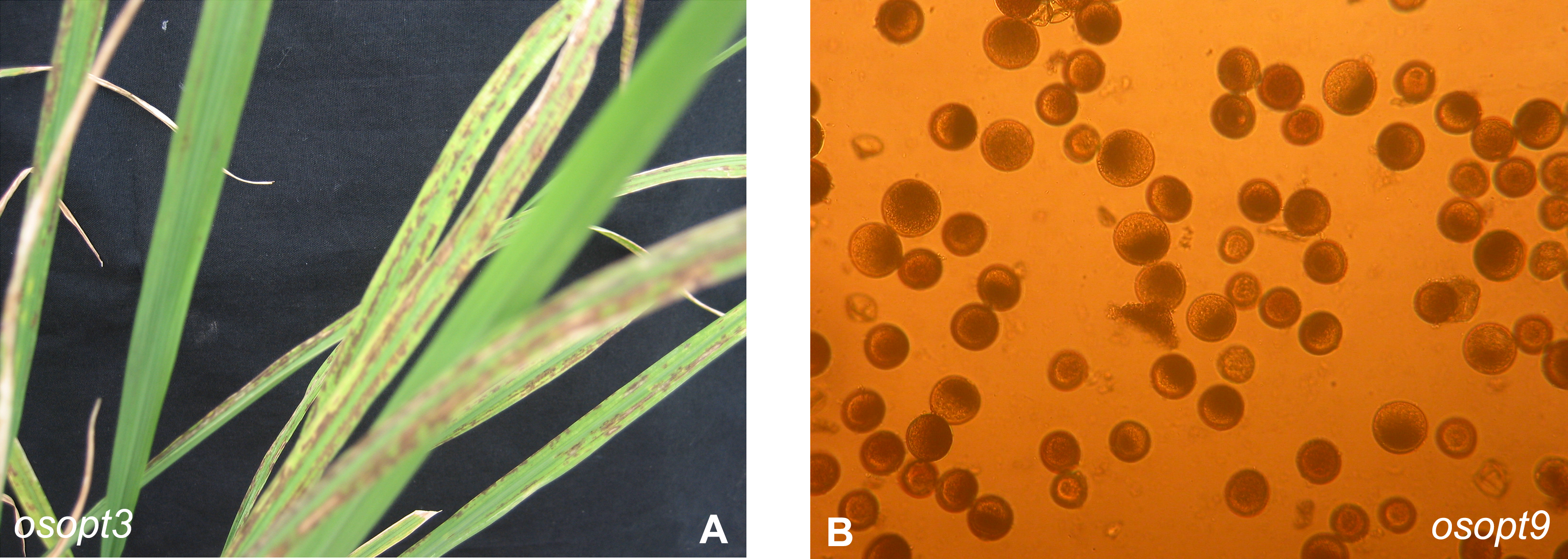

Supplement: Supplementary file 10 — Authors’ original file for figure 8 [file 12284_2012_10_MOESM10_ESM.tiff]
